# Active Smoking, Passive Smoking, and Risk of Nonalcoholic Fatty Liver Disease (NAFLD): A Population-Based Study in China

**DOI:** 10.2188/jea.JE20120067

**Published:** 2013-03-05

**Authors:** Yu Liu, Meng Dai, Yufang Bi, Min Xu, Yu Xu, Mian Li, Tiange Wang, Fei Huang, Baihui Xu, Jie Zhang, Xiaoying Li, Weiqing Wang, Guang Ning

**Affiliations:** 1 Key Laboratory for Endocrine and Metabolic Diseases of Ministry of Health, Rui-Jin Hospital, Shanghai Jiao Tong University School of Medicine, E-Institute of Shanghai Universities, Shanghai, China; 2 Shanghai Clinical Center for Endocrine and Metabolic Diseases, Shanghai Institute of Endocrine and Metabolic Diseases, Department of Endocrinology and Metabolism, Rui-Jin Hospital, Shanghai Jiao Tong University School of Medicine, Shanghai, China

**Keywords:** active tobacco smoking, passive tobacco smoking, fatty liver

## Abstract

**Background:**

The effect of active smoking on development of nonalcoholic fatty liver disease (NAFLD) is controversial, and there are limited clinical data on the relationship between passive smoking and NAFLD. We investigated whether active and passive smoking are associated with NAFLD.

**Methods:**

A total of 8580 subjects (2691 men) aged 40 years or older participated in a community-based survey in Shanghai, China. Information on active and passive smoking was collected using a validated questionnaire. NAFLD was diagnosed by abdominal B-mode ultrasound testing and serum liver enzymes.

**Results:**

NAFLD prevalence was 29.4% in never smokers, 34.2% in former smokers, 27.8% in light smokers (<20 cigarettes/day), 30.8% in moderate smokers (20–39 cigarettes/day), and 43.5% in heavy smokers (≥40 cigarettes/day). Fully adjusted logistic regression analyses revealed that, as compared with never smoking, former and heavy smoking were associated with increased risk of prevalent NAFLD, with odds ratios of 1.45 (95% CI 1.05–2.00) and 2.29 (95% CI 1.30–4.03), respectively. Active smoking and body mass index (BMI) had a synergistic effect on the risk of prevalent NAFLD; the combination of these risk factors was associated with the highest observed odds ratio for NAFLD: 8.58. In never-smoking women, passive smoking during both childhood and adulthood was associated with a 25% increase in the risk of prevalent NAFLD (OR = 1.25, 95% CI 1.05–1.50) as compared with no passive smoking.

**Conclusions:**

Passive smoking and heavy active smoking are associated with prevalent NAFLD in middle-aged and elderly Chinese. Active smoking and BMI have a synergistic effect on prevalent NAFLD.

## INTRODUCTION

Nonalcoholic fatty liver disease (NAFLD) is the most common chronic liver disease in Western countries, due to the high prevalence of obesity. NAFLD is characterized by fat deposits in liver cells and encompasses a spectrum of liver diseases, from simple steatosis to nonalcoholic steatohepatitis, liver fibrosis, and, ultimately, hepatocellular carcinoma. In recent years, Westernization of lifestyles has markedly increased NAFLD incidence among “lean” Asians, with the mean prevalence in China reaching 10%.^1^ NAFLD is associated with type 2 diabetes and cardiovascular disease (CVD) and now places a heavy economic burden on health care systems worldwide.

Active tobacco smoking is one of the most important environmental risk factors for chronic diseases such as cardiovascular disease, cancer, and type 2 diabetes.^2^^–^^4^ Recent studies suggest that tobacco use is also associated with increased prevalence and incidence of liver diseases.^5^^,^^6^ Smoking was an independent risk factor for advanced fibrosis in patients with primary biliary cirrhosis^5^ and patients with hepatitis C.^6^ Furthermore, Azzalini et al showed that cigarette smoking accelerated NAFLD progression in rats fed a high-fat diet.^7^ These studies provide evidence of a possible association between active smoking and NAFLD development. However, the clinical relevance of these experimental findings is controversial. A cross-sectional study reported that active smoking was associated with fibrosis in patients with biopsy-diagnosed NAFLD,^8^ while another study showed no relationship between active smoking and NAFLD.^9^

Clinical data on the relationship between passive smoking and NAFLD are limited. Therefore, we evaluated the effects of active and passive smoking on NAFLD prevalence in Chinese.

## METHODS

### Study design and population

We conducted this cross-sectional survey between March and August 2010 in the Jiading District of Shanghai, China. The design and protocols of the study have been previously described.^10^ Briefly, 10 375 subjects aged 40 years or older were invited and participated in a health examination. We used a standard questionnaire to collect information on lifestyle, medical history, and medication use and performed hepatic ultrasound examinations. Individuals with no information on smoking history (*n* = 31) or liver ultrasound findings (*n* = 43) were excluded, as were those with high alcohol consumption (>140 g/week for men; >70 g/week for women; *n* = 984), those seropositive for hepatitis B surface antigen (HBsAg; *n* = 371), those with a history of schistosomiasis, viral hepatitis, hepatic cirrhosis, liver carcinoma, or autoimmune liver disease (*n* = 282), and those with alanine aminotransferase (ALT), aspartate aminotransferase (AST), or γ-glutamyl transferase (γ-GT) levels greater than 3 times the upper limit (*n* = 84). Ultimately, data from 8580 individuals were included in the analysis.

The study was approved by the institutional review board of Rui-Jin Hospital, Shanghai Jiao Tong University School of Medicine. Written informed consent was obtained from all participants.

### Collection of medical data and biochemical measurements

A half-day examination was performed, including an interview, anthropometric measurements, and biochemical tests. Information on education status, physical exercise, and medical history was obtained by face-to-face interview. Physical activity at leisure time was estimated by adding questions on frequency and duration of moderate and vigorous activities and walking to the short form of the International Physical Activity Questionnaire (IPAQ).^11^

Body height and body weight were measured with subjects wearing lightweight clothes, and body mass index (BMI) was calculated as body weight in kilograms divided by the square of the height in meters. Blood pressure was measured 3 times with an automated electronic device (Omron Model HEM-752 FUZZY, Omron Company, Dalian, China), with a 1-minute interval between measurements, after at least 5 minutes of rest in a seated position. The hepatic ultrasound examination was performed by 2 experienced ultrasonographers, using a high-resolution B-mode tomographic ultrasound system (Esaote Biomedica SpA, Italy) with a 3.5-MHz probe.

Fasting blood samples were obtained after an 8-hour fast. Fasting and 2-hour postload plasma glucose (FPG and 2-h OGTT PG) levels, fasting serum triglycerides (TG), total cholesterol (TC), low-density lipoprotein cholesterol (LDL-C), high-density lipoprotein cholesterol (HDL-C), ALT, AST, and γ-GT were determined using an autoanalyzer (Beckman CX-7 Biochemical Autoanalyzer, Brea, CA, USA). Serum fasting insulin concentrations were determined by electrochemiluminescence assay (Sangon Company, Shanghai, China).

### Definitions

Normal BMI was defined as a BMI less than 25 kg/m^2^, overweight was defined as a BMI of 25 to 29.9 kg/m^2^, and obesity was defined as a BMI of 30 kg/m^2^ or greater.^12^ Hypertension was defined as a systolic blood pressure of 140 mm Hg or higher, a diastolic blood pressure of 90 mm Hg or higher, or use of antihypertensive medication. Type 2 diabetes was defined as a FPG of 7.0 mmol/L or higher and/or a 2-h OGTT PG of 11.1 mmol/L or higher, or use of antidiabetic agents, according to the 1999 World Health Organization criteria.^13^ Dyslipidemia was defined as presence of at least 1 of the following: TG of 1.69 mmol/L or higher, TC of 5.20 mmol/L or higher, LDL-C of 3.37 mmol/L or higher, and HDL-C less than 1.04 mmol/L.^14^ In accordance with the 2010 Chinese Guideline on Diagnosis and Treatment of NAFLD, a diagnosis of fatty liver was based on the presence of at least 2 of the following 3 abnormal findings: diffuse increased echogenicity of liver relative to kidney, ultrasound beam attenuation, and poor visualization of intrahepatic structures.^15^

### History of active and passive smoking

Information on active smoking was obtained by asking about current and lifetime smoking habits, and about age at smoking initiation, number of cigarettes smoked daily, and age at smoking cessation. Current smoking was defined as regular cigarette smoking (duration >6 months) at the time of examination. Former smoking was defined as a history of smoking for longer than 6 months and no current smoking (ie, at the time of the survey).

Passive smoking was defined based on responses to questions on whether there were smokers living in the participant's family during childhood and whether there were smokers living in the participant's family, or present at the workplace, during adulthood. If they answered in the affirmative, further information was obtained on passive smoking during adulthood (total days of exposure per week, duration of exposure at home and work). Passive smoking during adulthood was defined as exposure to passive smoking more than once per week and for longer than 1 year. Childhood exposure was truncated at age 18 years, and exposure after this age was included in adulthood exposure.

### Statistical analysis

Statistical analysis was carried out with SAS version 9.2 (SAS Institute Inc, Cary, NC, USA). Due to the extremely low prevalence of active smoking in women (18 records), only data on men were used in the investigation of the association between active smoking and NAFLD. Enrolled men were categorized into 5 active-smoking groups: never smokers, former smokers, light smokers (<20 cigarettes/day), moderate smokers (20–39 cigarettes/day), and heavy smokers (≥40 cigarettes/day).

Analysis of variance (ANOVA) and the Student–Newman–Keuls (SNK) test (for continuous variables) and the chi-square test (for categorical variables) were used to compare baseline characteristics across the active-smoking groups. Multiple logistic regression analyses were used to investigate the association between active smoking and NAFLD. We also used multiple logistic regression analyses to evaluate the combined effects of active smoking and abnormal BMI (overweight/obesity) on NAFLD prevalence. The odds ratios (ORs) of NAFLD associated with passive smoking were evaluated in men and women with no history of active smoking. Adjusted confounders in the multiple logistic models included age, education level (low/intermediate/high), alcohol consumption (yes/no), physical activity (yes/no), obesity (yes/no), hypertension (yes/no), diabetes (yes/no), use of antidiabetic medication (yes/no), dyslipidemia (yes/no), and fasting serum insulin.

All significance tests were 2-tailed, and a *P* value less than 0.05 was considered to indicate statistical significance.

## RESULTS

### Baseline characteristics of 5 active-smoking groups in men

The clinical characteristics of the 5 active-smoking groups are shown in Table 1 (*n* = 2691). The prevalence of NAFLD in never smokers (*n* = 999), former smokers (*n* = 310), light smokers (*n* = 583), moderate smokers (*n* = 707), and heavy smokers (*n* = 92) was 29.4%, 34.2%, 27.8%, 30.8%, and 43.5%, respectively. Mean age and BMI; distribution of education level, alcohol use, and physical activity; and prevalence of hypertension and NAFLD significantly differed among the 5 active-smoking groups (*P* < 0.05 for all tests).

**Table 1.** Characteristics of male participants (*n* = 2691) included in analysis of active smoking, by smoking status

	Never smokers	Former smokers	Light smokers (<20 cigarettes/day)	Moderate smokers (20–39 cigarettes/day)	Heavy smokers (≥40 cigarettes/day)	*P* value
*n* (%)	999 (37.1)	310 (11.5)	583 (21.7)	707 (26.3)	92 (3.4)	—
Age (years)^a,b,c,d^	62.9 ± 10.5	61.0 ± 10.0	58.3 ± 10.2	55.8 ± 8.8	55.4 ± 7.4	<0.0001
BMI (kg/m^2^)^a,b^	25.0 ± 0.1	25.6 ± 0.2	24.7 ± 0.1	24.9 ± 0.1	25.3 ± 0.3	0.0008
Education level						<0.0001
Low	286 (28.7)	96 (31.0)	129 (22.1)	172 (24.6)	28 (30.4)	
Intermediate	602 (60.5)	196 (63.2)	419 (71.9)	495 (70.7)	62 (67.4)	
High	107 (10.8)	18 (5.8)	35 (6.0)	33 (4.7)	2 (2.2)	
Alcohol use^a,b,c^						<0.0001
Yes	235 (76.5)	128 (58.7)	229 (60.7)	283 (60.0)	30 (67.4)	
No	764 (23.5)	182 (41.3)	354 (39.3)	424 (40.0)	62 (32.6)	
Physical activity^b,c,d^						<0.0001
Yes	717 (71.8)	218 (70.3)	367 (63.0)	410 (58.0)	50 (54.4)	
No	282 (28.2)	92 (29.7)	216 (37.1)	297 (42.0)	42 (45.7)	
Hypertension^a,b,c,d^						<0.0001
Yes	596 (59.7)	161 (51.9)	263 (45.1)	265 (37.5)	33 (35.9)	
No	403 (40.3)	149 (48.1)	320 (54.9)	442 (62.5)	59 (64.1)	
Dyslipidemia						0.64
Yes	635 (63.6)	209 (67.4)	384 (65.9)	482 (68.2)	61 (66.3)	
No	364 (36.4)	101 (32.6)	199 (34.1)	225 (31.8)	31 (33.7)	
Diabetes^c^						0.05
Yes	227 (22.7)	70 (22.6)	110 (18.9)	123 (17.4)	22 (23.9)	
No	772 (77.3)	240 (77.4)	473 (81.1)	584 (82.6)	70 (76.1)	
NAFLD^d^						0.02
Yes	294 (29.4)	106 (34.2)	162 (27.8)	218 (30.8)	40 (43.5)	
No	705 (70.6)	204 (65.8)	421 (72.2)	489 (69.2)	52 (56.5)	

Abbreviations: NAFLD, nonalcoholic fatty liver disease; BMI, body mass index.

Values are means ± SD, or numbers (proportion). *P* values were obtained from the chi-square test (for categorical variables) or analysis of variance (ANOVA) and the Student–Newman–Keuls (SNK) test (for continuous variables).

^a^*P* < 0.05 for former smokers vs never smokers; ^b^*P* < 0.05 for light smokers vs never smokers; ^c^*P* < 0.05 for moderate smokers vs never smokers; ^d^*P* < 0.05 for heavy smokers vs never smokers.

### Association between active smoking and NAFLD in men

As compared with never smokers, the unadjusted ORs for NAFLD among light, moderate, and heavy smokers were 0.92 (95% CI 0.74–1.16, *P* = 0.49), 1.07 (95% CI 0.87–1.32, *P* = 0.53), and 1.85 (95% CI 1.20–2.85, *P* = 0.006), respectively. After further adjustments for age, education status, alcohol consumption, physical activity, obesity, hypertension, diabetes, use of antidiabetic medication, dyslipidemia, and fasting serum insulin, the ORs for NAFLD among the 3 current-smoking groups were 1.03 (95% CI 0.78–1.36, *P* = 0.82), 1.21 (95% CI 0.93–1.58, *P* = 0.19), and 2.29 (95% CI 1.30–4.03, *P* = 0.004), respectively. After adjustment for all confounders, former smoking was associated with an increased OR for NAFLD (OR = 1.45, 95% CI 1.05–2.00, *P* = 0.03; Table 2).

**Table 2.** Risk of prevalent nonalcoholic fatty liver disease associated with active smoking in men

	Model 1		Model 2		Model 3	
		
	OR (95% CI)	*P* value	OR (95% CI)	*P* value	OR (95% CI)	*P* value
Never smokers	1.00 (ref)	—	1.00 (ref)	—	1.00 (ref)	—
Former smokers	1.25 (0.95–1.63)	0.11	1.36 (1.00–1.86)	0.05	1.45 (1.05–2.00)	0.03
Current smokers						
Light smokers	0.92 (0.74–1.16)	0.49	0.88 (0.68–1.15)	0.34	1.03 (0.78–1.36)	0.82
Moderate smokers	1.07 (0.87–1.32)	0.53	1.02 (0.79–1.31)	0.89	1.21 (0.93–1.58)	0.19
Heavy smokers	1.85 (1.20–2.85)	0.006	1.76 (1.05–3.00)	0.03	2.29 (1.30–4.03)	0.004

Data are odds ratios (95% CI). Odds ratios (ORs) and *P* values were derived from logistic regression models.

Model 1: unadjusted model; Model 2: adjusted for age, education status, alcohol consumption, physical activity, obesity, hypertension, diabetes, use of antidiabetic medication, and dyslipidemia, based on Model 1; Model 3: further adjusted for fasting serum insulin, based on Model 2.

The risk of prevalent NAFLD among overweight/obese men was higher than among those with normal weight, although the *P* for interaction did not reach statistical significance (smoking groups × BMI categories, smoking groups, and BMI categories included in the logistic regression model). In men of normal weight, as compared with never smokers, the fully adjusted ORs for former, light, moderate, and heavy smokers were 1.36 (95% CI 0.77–2.40), 1.04 (95% CI 0.66–1.63, *P* = 0.86), 1.61 (95% CI 1.05–2.46, *P* = 0.03), and 2.40 (95% CI 1.00–6.35, *P* = 0.05), respectively. In overweight/obese men, as compared with normal-weight never-smokers, the fully adjusted ORs for never, former, light, moderate, and heavy smokers were 2.69 (95% CI 1.92–3.76, *P* < 0.0001), 3.65 (95% CI 2.37–5.62, *P* < 0.0001), 3.06 (95% CI 2.06–4.54, *P* < 0.0001), 3.04 (95% CI 2.08–4.44, *P* < 0.0001), and 8.58 (95% CI 3.76–19.55, *P* < 0.0001), respectively (Figure).

**Figure.**
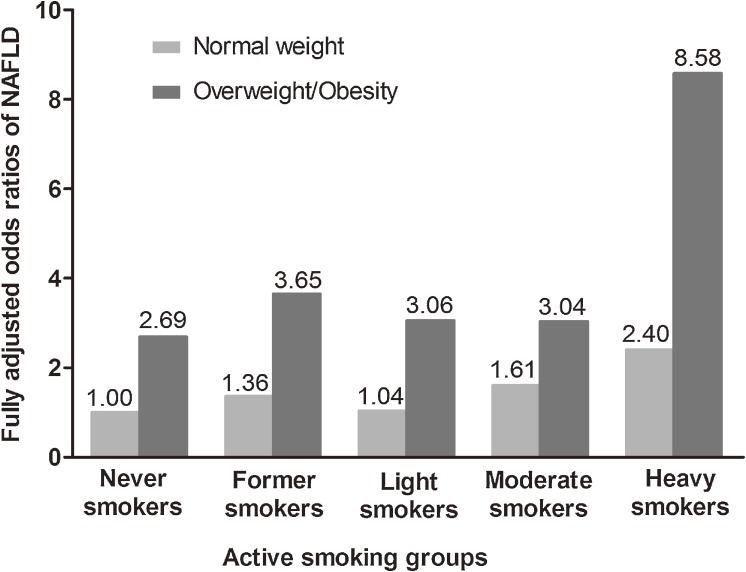
Combined effect of body mass index and active smoking on prevalent nonalcoholic fatty liver disease (NAFLD). Odds ratios (ORs) were adjusted for age, education status, alcohol consumption, physical activity, obesity, hypertension, diabetes, use of antidiabetic medication, dyslipidemia, and fasting serum insulin.

### Association between passive smoking and NAFLD in never smokers

The association between passive smoking and NAFLD was assessed in 6859 male and female never smokers (Table 3). Because of sex differences in NAFLD prevalence^16^ and the percentage of passive smokers in the population, the association was analyzed separately in men and women, although the interaction of passive smoking and sex was not statistically significant (*P* for interaction = 0.62). The prevalence of passive smoking was 33.4% during childhood and 13.9% during adulthood among men, and the respective values were 42.6% and 43.6% among women. In women, as compared with no passive smoking exposure, passive smoking exposure during both childhood and adulthood was associated with a significantly higher risk of prevalent NAFLD (OR = 1.25, 95% CI 1.05–1.50, *P* = 0.02) after controlling for possible confounders. However, in men, no significant association was found between passive smoking and NAFLD in childhood, adulthood, or both.

**Table 3.** Risk of prevalent nonalcoholic fatty liver disease associated with passive smoking (*n* = 6859)

	Cases	Participants	OR (95% CI)	*P* value
**Men**				
No passive smoke exposure	175	581	1.00 (ref)	—
Exposure during childhood only	77	254	0.90 (0.61–1.32)	0.74
Exposure during adulthood only	18	66	0.66 (0.33–1.29)	0.32
Exposure during both childhood and adulthood	20	70	0.88 (0.46–1.67)	0.88
**Women**				
No passive smoke exposure	651	2169	1.00 (ref)	—
Exposure during childhood only	315	1095	1.03 (0.85–1.24)	0.36
Exposure during adulthood only	342	1166	1.10 (0.91–1.33)	0.91
Exposure during both childhood and adulthood	426	1380	1.25 (1.05–1.50)	0.02

Data are odds ratios (ORs) and 95% CIs. *P* values were obtained by logistic regression models adjusted for age, education status, alcohol consumption, physical activity, obesity, hypertension, diabetes, use of antidiabetic medication, dyslipidemia, and fasting serum insulin. Regarding data on childhood passive smoking, there were 28 missing values among men and 50 missing values among women.

## DISCUSSION

We observed a positive association between heavy active smoking and NAFLD in a large, community-based population in China. In addition, tobacco smoking and BMI had a synergistic effect on NAFLD prevalence. Finally, exposure to passive smoke during both childhood and adulthood was associated with NAFLD prevalence in never-smoking women. To our knowledge, this is the first clinical study of the relationship between passive smoking and NAFLD.

Findings from animal studies suggest that tobacco use accelerates fat deposition in liver^7^ and that there is an underlying relationship between active smoking and NAFLD; however, epidemiologic studies have yielded conflicting results.^1^^,^^9^^,^^17^ In the present study heavy smoking was associated with higher risk of NAFLD, after adjustment for possible confounders. In addition, the prevalence and odds ratios of NAFLD gradually rose as the number of cigarettes smoked per day increased in current smokers, which suggests a dose-response relationship between active smoking and NAFLD. Indeed, numerous epidemiologic studies have reported that although the effects of light and heavy smoking on the risk of chronic diseases were not statistically significant, there was a dose-response effect with active smoking.^18^^,^^19^ Future studies should explore the associations of duration and quantity of active smoking (separately or in combination) with NAFLD prevalence. Such studies should address these questions in women, as these topics could not be analyzed in the present study due to the limited number of female active smokers.

Smoking was identified as a cofactor of obesity in NAFLD pathogenesis in animal and clinical studies.^7^^,^^20^ However, our study showed that moderate and heavy smokers of normal weight had a higher risk of prevalent NAFLD, demonstrating an independent relationship with BMI. The antiestrogenic effect of cigarette smoking, leads to changes in body fat distribution,^21^^–^^23^ which could potentially explain the independent role of BMI in the association of active smoking and NAFLD. Additionally, the increase in the risk of prevalent NAFLD among moderate smokers of normal weight was comparable to that in the general population (OR, 1.61 vs 1.21). Thus, smokers of normal weight, who may not be evaluated for NAFLD, deserve more attention with regard to NAFLD prevention. Furthermore, we found a much higher risk of prevalent NAFLD in overweight/obese light, moderate, and heavy smokers than in comparable smokers of normal weight. Hence, active smoking and BMI had a synergistic effect on NAFLD prevalence; the combination of these risk factors was associated with the highest observed OR for NAFLD: 8.58.

Exposure to passive smoke appeared to accelerate liver steatosis in mice,^24^ which suggests an association between passive smoking and NAFLD. We reported that passive smoke exposure during both childhood and adulthood was associated with increased NAFLD prevalence in women but not in men. We suspect that the small number of never-smoking men markedly decreased the statistical power to detect such an association. Furthermore, lack of information on passive smoking exposure in public places^25^ such as restaurant, bars, and card rooms, which was also a consideration in women, might have affected the statistical power. Such exposures need to be investigated further.

The odds ratios of NAFLD were higher in passive smokers than in light and moderate smokers. Information on the families of participants was not collected in the survey. Therefore, we cannot rule out the possibility that women exposed to passive smoking were family members of male heavy smokers. In addition, we cannot conclude that the effects of passive smoking on NAFLD prevalence were greater than those of light and moderate smoking, because the ORs of NAFLD associated with active and passive smoking were evaluated in different populations (men and women, respectively). Previous studies showed that exposure to sidestream smoke (the main component of passive smoking) yielded higher concentrations of harmful components than did mainstream smoke (the bolus of smoke inhaled by active smokers).^26^ However, there are few data directly comparing the effects of active and passive smoking on NAFLD.

Some limitations in the present study should be noted. First, this was a cross-sectional study and thus cannot establish temporal relations. Second, although liver biopsy is the standard criterion for NAFLD diagnosis, it is difficult to apply in community-based epidemiologic research. Furthermore, liver ultrasound is universally accepted in NAFLD diagnosis, due to its high sensitivity and specificity.^10^^,^^27^ Third, reported information on smoking should be corroborated by assessment of cotinine levels.^28^ Last, although we adjusted for known confounders of the association between smoking and NAFLD, we cannot rule out the possibility that there are unrecognized confounders.

In conclusion, passive smoking and heavy active smoking were significantly independently associated with NAFLD in middle-aged and elderly Chinese. Active smoking and BMI had a synergistic effect on NAFLD prevalence. Because tobacco smoking and overweight/obesity are relatively common in the general population, programs that encourage avoidance of these risk factors might reduce the likelihood of chronic diseases and mortality. Further prospectively designed population studies and clinical trials are needed to clarify the roles of active and passive smoking in NAFLD progression and prevention.
